# Exploring the dietary changes and support required for healthy eating with female students at UK universities: Findings from focus group discussions

**DOI:** 10.1371/journal.pone.0319388

**Published:** 2025-04-10

**Authors:** Eve F. A. Kelly, Merve Guney-Coskun, Michelle Weech, Rosalind Fallaize, Faustina Hwang, Julie A. Lovegrove

**Affiliations:** 1 Hugh Sinclair Unit of Human Nutrition, University of Reading, Reading, United Kingdom; 2 Department of Nutrition and Dietetics, Institute of Health Sciences, Istanbul Medipol University, Istanbul, Turkey; 3 School of Health, Medicine and Life Sciences, University of Hertfordshire, Hatfield, United Kingdom; 4 Biomedical Engineering Section, School of Biological Sciences, University of Reading, Reading, United Kingdom; United Arab Emirates University, UNITED ARAB EMIRATES

## Abstract

Students’ diets often change when leaving home and starting university due to increased responsibility for their diet and finances. However, there is limited qualitative research with students at UK universities about how their diets change during the transition to, and whilst at university and the reasons for these changes. The aim of this study was to qualitatively explore three topics: 1) specific dietary changes reported by students at UK universities, 2) reasons for these dietary changes and 3) how students can be supported to eat more healthily. Fifteen students (100% female, 54% white) across different academic years (60% undergraduate and 40% postgraduate) from the Universities of Reading and Hertfordshire were recruited. Four online focus groups were conducted, ranging from groups of 2 to 6 participants, using a semi-structured topic guide. Discussions were recorded and professionally transcribed. Transcripts were coded and themes derived for each research topic using qualitative analysis software. After joining university, dietary changes commonly reported by the students included either increased or decreased fruit and vegetable intake, increased snacking behaviour, and increased alcohol and convenience food consumption. Common reasons for changes included limited budget, time management struggles, a lack of cooking skills, and peer influence. Students suggested that reduced cost of healthy foods on campus and cooking classes to learn new skills could help them to adopt a healthier diet. These suggestions could be used to guide future healthy eating interventions for university students.

## Introduction

Student numbers at UK universities have steadily increased since the 1990s, reaching 2.86 million students in the 2021-22 academic year [1]. Notably, the proportion of 18 year olds remaining in higher education and going to university has risen from 25.0% to 35.8% in just over a decade [1]. Starting university is a time of large adjustment for most students, many of whom leave home for the first time. This period is associated with increased independence and responsibility for their own health and wellbeing [2]. A key aspect of optimal health and wellbeing is favourable dietary behaviour, which requires university students to effectively manage their grocery budget and take responsibility for purchasing healthy food and drink options [3]. However, research suggests that this population may have other priorities and face barriers to healthy eating [3–5]. Whilst there have been studies conducted in university populations worldwide, including the US and some European countries, there is a need for further research in students at UK universities due to cultural differences and the recent UK cost-of-living crisis.

Emerging evidence suggests a shift in dietary behaviours during the transition to university. These changes are often maintained by students during their time at university and can continue into later life [6]. Studies around the world report increased alcohol consumption [7,8], reduced fruit and vegetable intake [9] and a higher consumption of fast and convenience foods [10] in university students compared with their pre-university diet. Unfavourable dietary behaviours such as these whilst at university can lead to weight gain [7,11–14], lower academic performance [15], poorer mental health [16], sustained unfavourable dietary behaviours throughout life [6,17] and an increased longer-term risk of non-communicable diseases (NCDs) [18].

Specifically, a systematic review of the association between diet quality and mental health of university students demonstrated that unhealthy diets were associated with poorer mental health including depression, anxiety, and stress [16]. Additionally, poor dietary behaviours whilst at university are associated with lower academic performance in first year university students, particularly in males [15]. On the other hand, favourable dietary behaviours (e.g., breakfast consumption) and higher diet quality have been linked to improved academic performance [19,20].

A number of factors can influence students’ dietary choices including time constraints, social influence, a lack of nutrition/cooking knowledge, and/or limited kitchen facilities [3,4]. Limited budget also acts as a barrier to healthy eating, with around 1.5 million UK university students relying on student loans [21]. The current cost-of-living crisis in the UK (which started in 2021) is having an additional impact on many students’ dietary choices and includes students missing meals to keep food costs down [22]. A recent analysis of UK food prices reported that “less healthy” foods (classified using a nutrient profiling model) typically cost £0.33/100 kcal, whereas “more healthy” foods were almost 2.5 times more expensive (£0.81/100 kcal) [23]. Likewise, a meta-analysis suggested the daily cost of consuming a healthier dietary pattern was approximately US$1.50 (£1.17) more per 2000 kcal than a less healthy dietary pattern [24]. As such, students may opt for less healthy foods (such as those higher in salt, sugar and saturated fat) due to their lower cost compared with healthier food and drink items (such as fruit and vegetables, fish and poultry) [25].

Therefore, the aim of this study was to better understand any dietary changes that students experience when starting university in the UK, the reasons for these changes, and finally, how students can be supported to eat more healthily. These were qualitatively explored via discussions with students from UK universities to provide in-depth conversations and the sharing of experiences and ideas.

## Methods

### Ethics and consent

Favourable ethical opinion for conduct was given by the University of Reading’s School of Chemistry, Food and Pharmacy Research Ethics Committee (study number: 41/2022). After reading the Participant Information Sheet outlining the purpose of the study, what would be required of them if they took part, and any risks and benefits associated with the study, all participants provided digital consent by ticking each consent statement when registering for the study. At the start of each focus group, participants were reminded about the study procedure and given an opportunity to ask questions. They were then asked to re-confirm their consent verbally, which was captured on the Microsoft Teams recording and witnessed by the researchers.

### Recruitment and eligibility

This study aimed to conduct three focus groups each consisting of between 6-8 participants, which was in line with recommended sample sizes for focus groups [26–28]. Participants were recruited between the 5^th^ and 14^th^ of June 2023 from two UK universities, University of Reading and University of Hertfordshire, both of which offer a range of degree programmes to both undergraduate and postgraduate students. Students were informed of the study via university student mailing lists and advertising distributed around these campuses.

Eligibility criteria included being aged 18 years and over, studying full time in any academic year on a taught or research programme at the Universities of Reading or Hertfordshire, solely responsible for their own meal preparation and/or food purchases (fully catered students were also eligible on the basis they had freedom of choice in university canteens), and willing to discuss the focus group topics in a group setting. Both males and females were eligible to take part. Exclusion criteria included being pregnant, having a self-reported medical condition which significantly affected food choices or a diagnosed eating disorder, following a restrictive or weight loss diet, receiving dietary advice from a nutritionist or healthcare professional and/or being unable to contribute to a discussion spoken in English.

### Study design

Students interested in taking part in the focus group were invited to complete an online screening form (Online Surveys, Jisc, UK) to determine study eligibility. Participants were also asked to provide their ethnicity, year of study, degree course, and to rate how important a healthy diet was to them on a scale of 0 to 10 (with 0 being “not important” and 10 being “very important”). Students were not asked whether they were a home or international student at screening; however, some mentioned this during the discussions.

To encourage participants to share their opinions and perspectives with their group and feel at ease, they were allocated to a focus group with participants in a similar year of study. We used a qualitative exploratory approach [29] to gain further insight and understanding into the dietary behaviours of students since starting at a UK university. The researchers created a semi-structured topic guide for the focus groups (Table 1), with the topics, questions, and structure of the focus groups guided by previous literature, [4,30,31] which was used as a framework to develop the overarching research topics. Questions were designed to cover three main research topics (RT): 1) changes to students’ diets since starting university in relation to their pre-university diet (such as when living at home with their families), 2) reasons for these dietary changes and 3) support that could encourage the students to eat healthier. The latter included the type of support that the universities could provide, the optimal time to provide support, and barriers that may prevent student engagement with any support offered. This initial exploratory research aimed to develop ideas and find solutions to encourage healthier eating in university students. These could be used to direct the design of quantitative dietary studies and interventions.

**Table 1:** Focus group topic guide.

Topic goal	Question
Icebreaker for participants	1. Please share your name, degree course, year of study and one brief reason why you chose your degree course
Dietary changes since starting university	2. Thinking back to when you started university, can you describe any significant changes you noticed to what you had been eating and drinking prior to university? 3. What factors do you think influence(d) these changes?
Role of the university in healthy eating	4. Do you think the university can support students to eat healthily? 5. What could the university do to support students like yourselves to eat better whilst at university?
Support required to eat healthier whilst at university	6. Thinking about your own experience, when do you think university students have the biggest need for support to make healthy eating choices and why do you think this? 7. If the support that you have just discussed became available, would you be interested in this? 8. Can you think of any barriers that might prevent you from taking up any support offered?
Additional comments	9. Is there anything else you would like to say about your experiences or healthy eating whilst at university?

The focus groups took place after the end of the summer term (late June 2023) and were conducted online via Microsoft Teams. Discussions were led and moderated by one of two female researchers (MG (PhD student) or MW who was experienced conducting online discussions (Postdoctoral researcher)). The first author (EK), a female PhD student, acted as an observer who took summary notes for each discussion point and benefitted from familiarisation of the focus group discussions prior to data analysis. EK was muted during the focus groups to avoid overwhelming the discussion with multiple researchers. This study is part of her doctoral thesis. Video recording was used to facilitate conversation among the groups, to show non-verbal cues from participants (e.g., nodding in agreement), and to aid transcription (e.g., to identify which participant was speaking). The researchers did not know the participants prior to the study and therefore introduced themselves to each group, which included their role within the study and at the university. Participants were reminded that the researchers were keen to hear their honest opinions and experiences, including both positive and negative.

Furthermore, participants were asked to introduce themselves to the group (icebreaker), after which the three main RT were asked in turn, with the moderator introducing each individual question and asking the participants to share their thoughts (Table 1). Questions were open-ended to prevent researchers from influencing the participants responses. Participants who were not forthcoming with responses or who provided behavioural responses (such as nodding their head in agreement with another participant’s comments) were prompted to share their opinions. Occasionally, the moderator asked additional questions and/or provided prompts to obtain further insight and detail about the topics covered. At the end of each focus group, participants were asked whether there was any further information they would like to provide or expand upon. No repeat interviews were conducted. After completion, participants were given £10 for participating in the study.

The team of authors brings diversity of academic expertise, cultural and ethnic backgrounds, and individual university experiences to the study. The team collaborated closely throughout the study, so all stages of the research were shaped by these diverse perspectives. All authors had particular research interests in designing interventions to support individuals to eat more healthily.

### Data analysis

Microsoft Excel was used to calculate descriptive statistics from data collected at screening to characterise the focus group samples. All focus group recordings were sent to an independent professional company (Way With Words Limited, UK) for transcription. Transcripts were not returned to participants for comment or correction. Each transcript was read in full to re-familiarise the lead researcher (EK) with the content prior to analysis. The transcripts were then uploaded to the qualitative software NVivo 12 Pro (QSR International, 2017). Inductive and deductive thematic analyses were used to analyse the data by developing the themes based on the three RTs from the discussions. Firstly, each RT within each focus group transcript was individually coded (by EK) with codes assigned to recurring instances of similar quotes. For each RT, codes from all four focus group transcripts were combined into a single dataset. Codes with similar meanings were grouped together to form sub-themes which were further grouped to form overarching themes. As no new themes were derived by the end of the coding process, it was determined that data saturation had been reached and additional focus groups would not draw out new themes. Before being finalised, the proposed themes were discussed between all authors who all feel comfortable sharing their ideas between the group and come from a range of cultural backgrounds and prior university experiences. This brought diverse perspectives to the discussion. Quotations from the transcripts are presented in the results illustrating the themes within each RT. The Consolidated Criteria for Reporting Qualitative Research (COREQ) checklist [32] and a guide to reporting thematic analysis [33] directed the reporting of results.

## Results

### Sample characteristics

A total of 23 students registered their interest for the study; however, 8 participants failed to respond after screening or were unable to commit to the study. Therefore, four semi-structured discussions were conducted involving a total of 15 participants. The final size of the discussion groups ranged from 2 to 6 participants. All participants were university students from the Universities of Reading and Hertfordshire. Participant group characteristics are described in Table 2. Even though both males and females were eligible to participate and registered for the study, all participants who took part were female (*n* = 15). In line with the student body populations at the Universities of Reading and Hertfordshire (63%/61% undergraduates, 37%/39% postgraduates, respectively [34,35], these focus groups included a similar ratio of 1^st^ year undergraduates (UG1, 33%), 3^rd^ year undergraduates (UG3, 27%), and postgraduate students (40%). The majority of participants were studying a Psychology (60%) or Nutrition/Food (27%) related course. The mean self-rated importance of a healthy diet was 7 out of 10 (SD ± 1, range: 5-10), with 10 being “very important”.

**Table 2:** Characteristics of focus group participants (n = 15).

Characteristic	*n* (%) or mean (±SD)
**Sex: female (%)**	15 (100)
**Student status (%)**	
Undergraduate	9 (60)
1^st^ year undergraduate	5 (33)
2^nd^ year undergraduate	0 (0)
3^rd^ year undergraduate (or above)	4 (27)
Postgraduate	6 (40)
**Study discipline (%)**	
Psychology	9 (60)
Nutrition/Food	4 (26)
Data Science	1 (7)
Languages	1 (7)
**Ethnicity (%)**	
White	8 (54)
Asian	5 (33)
Black	2 (13)
**Self-rated importance of a healthy diet** (out of 10)^a^	7 ( ± 1)

^a^Importance of a healthy diet was self-rated by the students on a scale of 0 to 10 with 0 being ‘not important’ and 10 being ‘very important’.

### Qualitative results

**Fig 1:**
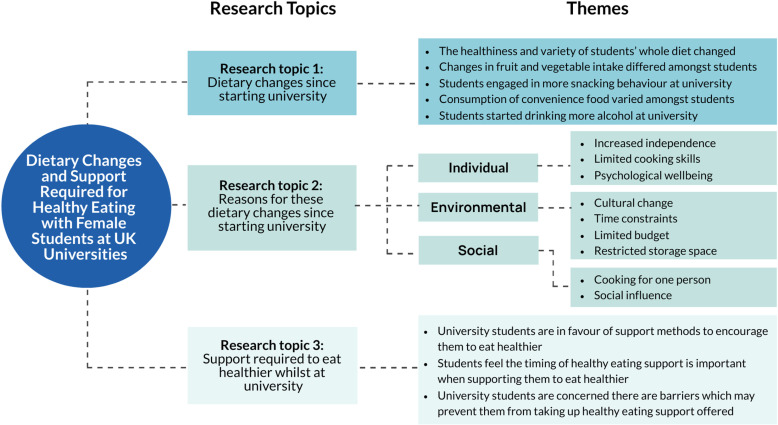
Summary of the themes that emerged from each of the three research topics (RT).

The mean focus group length was 89 minutes (SD ± 11, range: 76–102). The themes that emerged from each RT are summarised in Fig 1. Themes under RT2 were categorised by the researcher, based on the findings, into individual, environmental and social factors. Participants were not asked to provide feedback on these findings.

#### RT1: Dietary changes since starting university.

***The healthiness and variety of students’ whole diet changed:*** Compared with their pre-university diets (e.g., when living at home), students considered their overall diets to be less healthy whilst at university*: “I wouldn’t really eat as healthy as I would at home.”* (P7, UG1). They also reported eating less varied and simpler meals at university: *“I’d have a lot more variety of food at home, because we have a lot more ingredients and my parents are cooking for four people”* (P9, UG1).

***Changes in fruit and vegetable intake differed amongst students:*** It was noted that fruit and vegetable consumption decreased for some participants whilst at university: *“So, I ate a lot more healthier at home, and then when I was at uni, I found it harder to eat vegetables and stuff because they go off so quick.*” (P9, UG1). In contrast, others experienced increased consumption: *“I’d just buy vegetables and just fry them up. So, I eat more healthy when I was at university than [when] I was at home.”* (P6, UG1).

***Students engaged in more snacking behaviour at university:*** Some students mentioned an increase in snacking behaviour during their time at university: *“I think a lot of snacky things as well, because sometimes, at the end of the day, after university, you don’t want to come home and cook for yourself.”* (P6, UG1) and *“I’d always just be snacking all the time.”* (P7, UG1)*.*

***Consumption of convenience food varied amongst students:*** Some students discussed consuming more convenience foods such as takeaways, fast food, and ready meals at university*: “When you go out clubbing, it’s a tradition to get a takeaway afterwards, so I think a lot of people do that.”* (P6, UG1). However, some students said they avoided such foods due to their cost and portion size: *“I mostly avoid takeaway food because it’s very expensive and the quantity is less.”* (P14, PG).

***Students started drinking more alcohol at university:*** Increased alcohol consumption was also experienced by some students whilst at university: *“I didn’t really start drinking socially until I came to university”* (P3, UG3) and *“At home I wouldn’t really drink…but with uni, especially in the UK, there’s a drinking culture. I definitely drank a lot more.”* (P4, UG3). 

#### RT2: Reasons for these dietary changes since starting university.

***Individual factors:***

**Increased independence.** The increased independence and freedom experienced by many students at university was linked to changes in their dietary behaviour. Some students highlighted the positive effect of increased independence on their diet: *“I think, at the beginning, at university, I started eating healthier because at home I can’t really choose what to eat, and sometimes when I want something light I go for a salad.”* (P4, UG3). However, other students reported that increased independence negatively affected their diet: *“Being at university it kind of meant that I went a bit crazy with freedom and I’d order lots of takeout food a bit more often.”* (P3, UG3).

**Limited cooking skills.** Some students acknowledged that they did not cook much before starting university: *“I was living with my family and I was not cooking much.”* (P5, PG). Others suggested that many students lack cooking knowledge and skills: *“Most of my flatmates didn’t know how to cook…If you’re in halls and they’re not catered, you’re going to be cooking frozen pizzas because you don’t know how to cook.”* (P1, PG).

**Psychological wellbeing.** Extra pressure and stress from university and exams also contributed to changes in the students’ dietary behaviours. For some students, stress resulted in decreased appetite: *“I noticed that I lost a lot of weight since I came here because I tend to lose a lot of weight when I get stressed, and I tend to lose my appetite as well.”* (P12, PG). Others described how they noticed their friend’s diets had changed during exam periods: *“I know some of my friends, they said that they started eating more because there were snacks during revising and stuff like this, and that they gained weight, perhaps, or stuff during exam season, and were stressed about that.”* (P8, UG1).

***Environmental factors:***

**Cultural change.** Some participants mentioned they had moved to the UK for their studies and spoke of dietary changes due to the lack of availability of foods that were familiar to them: *“I’m from a different country, so when I started, obviously, it was basically changing the whole diet because what is readily available in my country is not what’s readily available in the UK.”* (P15, PG). Additionally, the change in culture enabled students to adapt their food choices and preferences: *“At home I would mainly have an East Asian diet. Meaning, rice as the main source of carbs but I’m not actually a huge fan of rice, I like pasta more. So when I came to uni, actually, my whole taste becomes a lot more European. And I think also the groceries available here in this country is very different to things available at home.”* (P4, UG3).

**Time constraints.** Students spoke about struggling to balance the time required to prepare and eat regular meals alongside their university and employment commitments: *“But nowadays because the lecture times are at different times or I might have work at a certain time, I find it a lot harder to regulate how to have three meals a day.”* (P3, UG3) and *“Before my PhD I had more of a nine to five job, so it was easier to plan meals as well.”* (P1, PG). Limited time to prepare and eat meals became more of a problem when students were stressed or had exams: *“The time when there is a lot of exams and deadlines is the time where I give more importance to that, so it’s not really my choice of what I want to eat.”* (P12, PG) and *“Especially during studying for exams as well, I would say that I felt like I had less time to eat and was more focused on my exams.”* (P10, UG3).

**Limited budget.** Participants across the academic year groups spoke about the impact of a limited budget in relation to buying healthier foods, eating three meals a day, and food choice. P8 (UG1) stated that *“Sometimes healthier foods are more expensive. Even fruits and vegetables can be more expensive than [instant noodle brand], which can be 40p, do you know what I mean? So, I think some people would just prefer to eat something cheaper and eat something than pay two, three times more to eat something healthier.”* This was supported by others: *“Also, just affording three healthy meals a day is something that is quite expensive and can be considered a luxury, I think, to many students... The cost-of-living crisis is huge right now and a lot of people are struggling to figure out how to eat healthily.”* (P3, UG3), *“I think there’s definitely a drive that some students have to make an effort to have healthy meals and prepare those things, but ultimately, you’re kind of bound by your student budget.”* (P10, UG3) and *“I tend to go for the food that is most convenient as well as cheap”* (P12, PG). One student also mentioned the cost of foods they would typically have in their home country were less expensive than in the UK and described the subsequent impact this had on their diet, *“Here, the vegetables that are from India are very expensive, so we started eating vegetables that are available here and the variety of vegetables reduced.”* (P14, PG).

**Restricted storage space.** Some students mentioned limited food storage space in their shared accommodation influenced the quantity of food they bought. *“Because we’d obviously only have one fridge shelf. So, it was limited what you could get, because obviously otherwise you couldn’t store it.”* (P7, UG1). Limited storage space also influenced the type of food bought, such as buying fresh vs frozen fruit and vegetables: *“I might choose to buy a bag of frozen vegetables rather than having fresh vegetables in my fridge because I need space for butter and bread and other things in my fridge.”* (P3, UG3). In accommodation where there was more food storage and cookware space enabled students to have more choice over what they cooked: “*I’ve actually been living with just one other person, and I’ve just really noticed that I have been making better decisions. And I have been able to buy bulk and store it and make meals for the week and take my time in the kitchen and have more than one saucepan.*” (P10, UG3).

***Social factors:***

**Cooking for one person.** Many participants spoke about the challenges of cooking for one person: *“Cooking for one person can be really annoying and difficult because you can’t really buy that amount of food.”* (P11, UG3). This also had an impact on the variety of both the undergraduate and postgraduate students’ diets: *“I’d cook one meal, and then I’d have that same meal every day for the whole week.”* (P7, UG1) and *“Whereas because now I live by myself, I normally batch cook which means that I end up eating the same meal, for example, the same lunch for a few days in a row.”* (P1, PG). Food waste when buying food and cooking for one person was also mentioned: *“If I’m just buying for me, then I’m not going to buy as much veg because it won’t get used and then it’s a waste of money.”* (P9, UG1).

**Social influence.** Participants reflected on the impact of peer influence and university culture on their increased alcohol intake and fast-food consumption: *“I definitely drank a lot more… and sometimes it just helps with the social situation.”* (P4, UG3) and *“Especially after the Student Union Wednesday, you would literally leave, and they [popular takeaway pizza company] would be right there with pizzas you could get. And obviously everyone’s leaving drunk so you’re just going to it then without thinking.”* (P8, UG1). 

#### RT3: Support required to eat healthier whilst at university.

***University students are in favour of support methods to encourage them to eat healthier:***Participants identified methods of promoting and engaging university students in healthier eating. One method was increasing the amount of healthier food options on campus: *“Maybe, a store with ready-to-make healthy food, maybe like sandwiches that are healthier or stuff like that.”* (P15, PG) and *“The food that they serve on campus, they could just incorporate more healthy options.”* (P13, PG). Other participants suggested lowering the cost of healthier food options: *“The healthier options are a bit more expensive for students. Maybe that could change, if they have any power on it, they could make more healthier options available and cheaper.”* (P5, PG) *and “Maybe, a grocery shop inside the uni, a cheap grocery shop. We do have one in the uni, but when we compare it to [budget supermarket chain] it’s expensive.”* (P14, PG). The idea of group cooking classes for students to learn new skills was also viewed favourably by participants: *“I think that would be super helpful for so many people, because I think so many people go, obviously, living at home for 18 years, having all their meals cooked for them, to then going to uni where you have to make every food for yourself.”* (P8, UG1). Providing students with recipes was also supported by many students: *“I think that is a very good idea, to have easier recipes with less ingredients, but are still healthy and incorporating a good balanced diet into them.”* (P9, UG1) and *“I think there’s a lot information around uni about alcohol consumption and being smart and safe about that. So, I think the same could be used for food and definitely recipes for people who have just started uni and maybe don’t know what’s cheaper and quick and easy to make.”* (P11, UG3).

***Students feel the timing of healthy eating support is important when supporting them to eat healthier:*** The start of university was identified by many participants as a good time to educate and support university students with healthy eating behaviours: *“It’s probably good to do it right at the beginning, because everyone’s a bit clueless.”* (P6, UG1) and *“The start of uni because it can set a good foundation. So, I really made an effort to eat healthily when I first started uni. And that helped me to build up from it for the rest of the uni years.”* (P4, UG3). In addition, students felt continued support throughout their time at university would be beneficial as well as during more stressful periods such as exam season: *“I just think it needs to be a constant message and it applies to all years at all stages of your course.”* (P10, UG3) and *“In exam season, it’s very easy to just get lost and forget what you’re doing.”* (P7, UG1).

***University students are concerned there are barriers which may prevent them from taking up healthy eating support offered:*** Although students recognised the need for healthy eating support whilst at university, some identified barriers which may prevent them from taking up support offered, such as a lack of time and potential costs associated with the support offered. *“I feel like the classes are a great idea. But there’s a few things that the uni runs that are good ideas but because there’s so much else going on, it’s hard to find the time to actually join them and partake in them.”* (P7, UG1) and *“So, as students, the cost is another thing, so if we’re having to pay an amount that’s quite expensive to learn to make one portion of meal I think that might kind of take away the point of it.”* (P4, UG3). One student mentioned that some students may feel ashamed to attend cooking classes: *“There could be people who might be a bit more shy and don’t want to go these sessions or might feel a bit ashamed.”* (P3, UG3). Another student mentioned their individual food preferences would affect their engagement with cooking classes: *“If it’s going to be something that I’m not used to eating, then I think I would not really participate.”* (P13, PG).

## Discussion

The focus group participants highlighted a variety of changes in their dietary behaviour since starting university and offered several explanations for these changes. They also identified barriers which prevented them from adopting healthier dietary behaviours; however, they were open to receiving healthy eating support.

Students mentioned changes to their whole diet since starting university and, for some, there was less variety in their diet. Reasons for these included difficulties in cooking for one person because ingredients are usually enough for more than one portion. Therefore, some students were eating the same meals multiple times a week to avoid food wastage although this may lead to a lack of diversity in the diet. Since diverse diets are generally more expensive [23–25], this may discourage students from increasing variety in their diets. For example, consuming five food groups a day (dairy, fruit, vegetables, meat, and grains) is associated with an 18% higher food cost than consuming three or less food groups a day [36]. A lack of diet variety has been found in other student populations such as in Mexico and Brunei [9,37], and particularly in those with specific dietary patterns such as snackers [38].

Some students reported eating fewer fruits and vegetables whilst at university. This is consistent with a self-reported health behaviours study in UK university students that found 86% of participants did not consume the UK’s recommended intake of 5 fruit/vegetable servings a day [8]. Since fresh foods, such as fruit and vegetables, have short shelf-lives, participants in the current study were cautious about how much food they were buying to avoid food wastage. Additionally, limited storage space in communal kitchens (e.g., university halls of accommodation) meant that students prioritised space for other fresh food products, with some choosing to buy frozen varieties instead. A lack of facilities and limited storage space in university accommodation has been well documented in other research looking at barriers to healthy eating in university students in the United States and Spain [39,40].

Some participants also reported increased alcohol consumption at university. Whilst alcohol consumption was not quantified in this study, other studies have also reported high alcohol intakes in university students, including UK students [41,42]. Although students’ alcohol consumption can be high across all years of study, this tends to reduce as students progress through university [43]. Students’ living environments, such as living on campus and having numerous room-mates, are also linked to drinking more alcohol [44]. Frequently consuming alcohol has been linked to lower academic performance in first year university students [15]. Concerningly, a systematic review of cohort studies identified that elevated consumption of alcohol during adolescence may continue into adulthood and lead to dependency [45], both of which increase the risk of cancer, diabetes, cardiovascular, liver and pancreatic diseases [46]. University culture and social influence were identified as contributors to increased alcohol consumption in this study, which supports other studies reporting peer pressure, a desire to enjoy an evening and social events centred around alcohol act as contributors to increased alcohol consumption in university students [43,47].

Other dietary changes included increased snacking and increased consumption of convenience foods, sometimes arising from limitations in cooking abilities. Sprake, et al. [48] found that students with limited cooking ability were less likely to have a healthier diet (defined as vegetarian or health-conscious dietary patterns) than students who could cook. Interventions targeting cooking ability could be interesting and worthwhile because better cooking ability is also associated with greater adherence to the Mediterranean diet [49], which is known for its health-promoting effects [50–52]. Therefore, improving cooking skills could positively impact dietary intake [53] and health outcomes in later life. Time constraints were also a barrier to eating healthily, where students reported challenges with time management to prepare meals alongside other pressures, such as socialising, coursework and exams. When these other factors took priority over healthy eating and spending time preparing meals, this sometimes resulted in students relying on foods that required little preparation. This supports previous research in other European countries which has consistently found time pressures at university negatively influence dietary choices, such as relying on fast and convenience foods [3,4,40].

The current cost-of-living crisis in the UK is considered “very concerning” for over 50% of university students, with 32% being very concerned about paying for groceries and food costs [54], and this was also a worry for our participants. Reports suggest that 1 in 4 UK students cannot afford food and regularly go without [55], which is supported by a Times Higher Education report that highlighted 63% of students spent less money on food, with 28% going to extreme measures, such as skipping meals, to save money [22]. Therefore, it was unsurprising that students in this study discussed the considerable influence their budget has on their dietary choices. They would also generally purchase cheaper less healthy options than more expensive healthier alternatives, supporting previous research that reported students relied on cheaper food with poorer nutrition [55].

Culture changes were also responsible for dietary changes in students who had relocated to the UK to study. Foods that international students would typically consume in their home country were either not available in UK food stores or more expensive, which meant that for some students, their whole diets changed. This has been identified by other groups investigating the dietary experiences of international students in the UK and US [56,57] and can lead to international students adopting the dietary practices of the host country (dietary acculturation) [58].

Interestingly, some students mentioned that their psychological wellbeing (e.g., high stress) negatively affected their dietary behaviour. However, poor diet quality has been shown to negatively impact mental health, which may subsequently reinforce poor dietary choices and poorer diet quality affecting their mental health even further [16]. For example, higher perceived stress in students has been significantly associated with infrequent fruit and vegetable consumption; however, consumption of such foods may protect against stress and anxiety [59], leading to a cyclical detrimental effect between mental health and dietary behaviour. Therefore, university students who have poor dietary behaviours and low diet quality may be negatively impacting their mental health, which is important given 57% of students in the UK reported having a mental health issue and 30% said their mental health had worsened since starting university [54].

Whilst many dietary changes were not conducive to a healthy lifestyle some students thought their diet was healthier after starting university because they were free to choose which foods to eat and in what quantities, such as wanting to eat more fruits and vegetables than what was available to them at home pre-university. This is consistent with previous research which found that not all students at UK universities had unhealthy dietary patterns and this was particularly likely in female students and those who had greater self-reported cooking ability [48].

Overall, the dietary changes discussed by the students should be confirmed in studies which accurately assess dietary intake to compare how university students’ diets have changed since starting university.

The student feedback highlighted that there are several opportunities to provide healthy eating support for university students. For example, a lack of cooking skills was identified as a barrier to healthy eating and students were in favour of introducing cooking classes to teach them new skills. The benefits of cooking skills were demonstrated in previous research which found that although individuals may wish to cook healthy meals, a lack of cooking skills can act as a key barrier to eating healthily [60]. Evidence also suggests that frequent food preparation and cooking in young adults is associated with a higher likelihood of meeting the dietary recommendations for fruit, vegetables and wholegrain intakes and decreased consumption of fast-food [61] as well as greater adherence to a Mediterranean diet [53]. Cooking skills interventions with various populations, including university students, have successfully improved efficacy and confidence in cooking ability and influenced positive dietary behaviours [62–64]. Therefore, motivating students to develop cooking skills may improve their confidence following a recipe, encourage them to prepare their own meals and improve diet quality [65].

Whilst introducing cooking classes may be beneficial, it is important to acknowledge that there are other factors which may influence student engagement with the support. Students mentioned that the cost and timing of support on offer may impact whether they are available to make use of it, particularly if they have other priorities. Therefore, alternative interventions, such as online courses, which are cheaper and allow students to engage with the material in their own time may be beneficial. This could also ensure the interventions are accessible to students who may feel ashamed about lacking cooking skills and would benefit from indirect healthy eating support. Educational interventions could include providing budgeting tips and recipe booklets to students to teach them how to eat well on a budget. Other suggestions from the students include improving storage space and cooking facilities in student accommodation and for universities to lower the cost of healthy food items on campus, which was also proposed by Sprake, et al. [48].

Students also considered the timing of support to be important as they have other commitments which may take priority. Some students felt that healthy eating support should be offered continuously throughout their time at university, whilst others felt it was most important during exam seasons and other stressful times of the year with deadlines. In general, research suggests that implementing dietary interventions during the transition of late adolescence to young adulthood could be beneficial to university students as leaving home is associated with poorer diet quality [66]. For example, students living away from home may be more likely to have unfavourable dietary behaviours (such as lower fruit and vegetable consumption) compared with students still living at home [67]. Therefore, supporting the transition of leaving home with healthy eating advice may be beneficial.

However, it is important to note that postgraduate students have typically been responsible for their dietary intake for longer than other students, such as first year undergraduates. Evidence suggests that older students may become more aware of the impact of their dietary choices on their health and positively change their eating behaviours to reflect this [48,68]. Therefore, the type of healthy eating support required by postgraduate students may differ from undergraduate students.

Overall, improving students’ dietary behaviour could have many potential benefits, including improved academic performance [19,20], reduced risk of poor mental health linked to dietary choices [16], and reducing the likelihood of sustained poor dietary behaviours throughout life [6].Therefore, given the importance of eating healthily at university, studies that evaluate student engagement with any form of dietary intervention and accurately assess changes in diet quality in response to these interventions are warranted.

## Strengths and limitations

This study is one of few to qualitatively explore the changes that students at UK universities make to their diets and the reasons for these changes. The findings are valuable for identifying opportunities for interventions that target these areas and are welcomed by the end-users. The study recruited British as well as international students and students from a range of academic years (including undergraduate and postgraduate), which resulted in a rich discussion amongst the participants. Additionally, the study was designed to group students in similar academic years together which can help encourage participants to feel confident voicing their opinions and experiences [69] and promote group interaction [70].

A limitation of this study is that, although both males and females were eligible to take part, all participants were female, so the results may have differed if male university students were included. For example, research suggests that women tend to be more concerned about making healthy dietary choices than men [71–74], which could also explain why this study received greater interest from women than men. Some of the discussions were small, consisting of two participants due to restrictions with participant availability as the academic term had finished for many students. However, there were beneficial insights from all discussions. Additionally, participants were not asked about their understanding of the term ‘healthy eating’ nor provided with a reliable definition during the discussions. Although most participants were enrolled on a health-related degree (psychology or a food/nutrition-based course), it could be assumed that these students had a greater interest and/or knowledge about diet and nutrition than the general student population; however, the focus groups showed these students still faced barriers with eating healthily whilst at university and were also open to receiving support. Assuming greater nutrition knowledge in this group, this may also explain why nutrition knowledge deficit was not identified as a barrier to healthy eating, contrary to other studies with university students [39,75].

Finally, the students were from one of two UK universities (Universities of Reading and Hertfordshire) and from a limited range of degree programmes on offer at both universities so the generalisability of this study’s findings to other UK student populations may be limited. Nevertheless, the results from this study still identify potential opportunities for improving dietary support for students at UK universities which could apply regardless of their sex, degree course or university location.

## Conclusions

In this study, the students reported a variety of dietary changes since starting university, including increased alcohol and convenience food consumption, less variety in their diet and a mixture of both increased and decreased fruit and vegetable intake. They identified that a lack of time, limited budget, cultural changes, and/or psychological wellbeing are some of the reasons responsible for these changes. The students were open to being offered support from their university to encourage them to eat healthier suggesting cooking classes and reducing the cost of healthy food options on campus. Future healthy eating interventions should target the barriers students face in following a healthy diet whilst at university, such as limited budget and time constraints. Finally, interventions should be optimally timed, such as when starting university and/or during exam periods, to encourage student engagement and provide support when it is most beneficial to them.
